# Physiological Reduction in Left Ventricular Contractile Function in Healthy Postpartum Women: Potential Overlap with Peripartum Cardiomyopathy

**DOI:** 10.1371/journal.pone.0147074

**Published:** 2016-02-09

**Authors:** Sitara G. Khan, Narbeh Melikian, Sitali Mushemi-Blake, William Dennes, Fadi Jouhra, Mark Monaghan, Ajay M. Shah

**Affiliations:** 1 Department of Cardiology, King’s College London British Heart Foundation Centre, London, United Kingdom; 2 Department of Cardiology, King’s College Hospital, London, United Kingdom; 3 Department of Obstetrics and Gynaecology, King’s College Hospital, London, United Kingdom; Temple University, UNITED STATES

## Abstract

**Aims:**

Peripartum cardiomyopathy is a potentially life-threatening cause of heart failure, commoner in Afro-Caribbean than Caucasian women. Its diagnosis can be challenging due to physiological changes in cardiac function that also occur in healthy women during the early postpartum period. This study aimed to (i) establish the overlap between normal cardiac physiology in the immediate postpartum period and pathological changes in peripartum cardiomyopathy ii) identify any ethnicity-specific changes in cardiac function and cardiac biomarkers in healthy postpartum women.

**Methods and Results:**

We conducted a cross-sectional study of 58 healthy postpartum women within 48 hours of delivery and 18 matched non-pregnant controls. Participants underwent cardiac assessment by echocardiography and strain analysis, including 3D echocardiography in 40 postpartum women. Results were compared with 12 retrospectively studied peripartum cardiomyopathy patients. Healthy postpartum women had significantly higher left ventricular volumes and mass, and lower ejection fraction and global longitudinal strain than non-pregnant controls. These parameters were significantly more impaired in peripartum cardiomyopathy patients but with overlapping ranges of values. Healthy postpartum women had higher levels of adrenomedullin, placental growth factor (PlGF) and soluble fms-like tyrosine kinase-1 (sFlt1) compared to controls. The postpartum state, adrenomedullin, sFlt1 and the sFlt1:PlGF ratio were independent predictors of LV remodelling and function in healthy postpartum women.

**Conclusion:**

Healthy postpartum women demonstrate several echocardiographic indicators of left ventricular remodelling and reduced function, which are associated with altered levels of angiogenic and cardiac biomarkers.

## Introduction

Peripartum cardiomyopathy (PPCM) is a potentially life-threatening cause of heart failure that manifests in the last month of pregnancy or the first few months following delivery.[1] Mortality rates range from 10–30%, and complete recovery of left ventricular (LV) function occurs in less than half of women.[1] There are significant geographic and ethnic variations in the incidence of PPCM, with especially high rates in South Africa and Haiti whereas the epidemiology in Europe is poorly characterised.[2] The underlying pathophysiological mechanisms of PPCM are incompletely understood. Recent studies have identified oxidative stress, inflammation and an imbalance of angiogenic factors in the post-partum period as potentially important factors.[3] A substantial proportion of PPCM patients present in the post-partum period when diagnosis may be difficult due to the physiological changes associated with delivery.

It is well recognised that the maternal heart undergoes significant structural changes during pregnancy, with the LV demonstrating features of a volume overload state.[4–6] However, most previous studies were performed using 2D or M-mode echocardiography, before the widespread availability of more advanced methods such as 3D echocardiography.[7] Furthermore, very limited data are available on cardiac function in healthy women during the early postpartum period. It is also not known whether cardiac function in this period differs by ethnicity.

This study aimed to establish the normal parameters of cardiac structure and function in healthy Caucasian and Afro-Caribbean women in the immediate postpartum period and to define the overlap between normal postpartum physiology and pathological changes in PPCM.

## Methods

### Study population

The study was performed between June 2012 and September 2013 at King's College Hospital in South London, which has a large maternity unit and an approximately equal number of Caucasian and Afro-Caribbean women admitted for delivery. The study complied with the Declaration of Helsinki and was approved by the London Wandsworth Research and Ethics Committee. All participants provided written informed consent.

We prospectively recruited 58 women (mean age 31.1±5.6 years) less than 48 hours postpartum following an uncomplicated vaginal delivery. Only subjects free from pre-existing medical conditions and on no regular medications were included, and all were non-smokers. All subjects underwent 2D and Doppler echocardiography while 40 of the 58 women also underwent 3D LV imaging. Five subjects had suboptimal apical echocardiographic views that precluded accurate 3D analysis, and 13 ended the study prior to 3D image acquisition due to an unsettled baby. All participants had blood pressure measured prior to the echocardiographic study. A subset of PP women (n = 30) provided blood samples for analysis of biomarkers. Six PP women with abnormal results had subsequent repeat echocardiography and blood tests. A control group of 18 healthy, age and ethnicity-matched women (mean age 32.1±7.2years), who had not been pregnant in the last year, underwent echocardiography and 14 of these consented to biomarker analysis.

Given the relatively low incidence of PPCM,[2] the prospective recruitment of participants was not feasible. Instead, we studied a retrospective sample of 12 consecutive patients diagnosed with PPCM over a 3 year period according to the criteria set out by Sliwa et al.[1]All patients were diagnosed post-partum (42.5 ±9.2 days after delivery) by a total of 4 different operators. Eleven had a 2D echocardiogram within 24 hours of presentation and the 12^th^ patient had echocardiography upon referral to the cardiac outpatient service. 3D echocardiography was performed between 2–7 days after the initiation of treatment in all patients.

### Echocardiography and Doppler studies

2D and 3D echocardiography was performed according to the American Society of Echocardiography and EAE/ASE guidelines.[8] An iE33 system (Philips Medical Systems, Andover, Massachussetts) was used with a 2.5-MHz transducer and anX5 matrix array transducer to acquire 2D and 3D images, respectively. Images were acquired in the left lateral decubitus position. The 3D examination was focused on the LV; a full-volume 3Ddataset was obtained from the apical window during a breath-hold, to minimise the risk of stitch artefacts. ECG-gated acquisition was used to combine real-time 3D data over four cardiac cycles.

Pulsed wave Doppler at the mitral leaflet tips on the apical 4-chamber view was measured to quantify early (E) and atrial (A) phases of diastolic filling. E’ measurements at the lateral and septal mitral annulus were obtained using tissue Doppler imaging (TDI), and the average of the two measurements was used as a measure of global LV diastolic function.[9] Continuous wave Doppler sampling at the aortic valve on the apical 5-chamber view was used to provide data on the timing of aortic valve opening and closure.

### Echocardiographic data analysis

Offline analysis was performed using Xcelera (Philips) and Qlab (Philips) software by two independent operators. LV volumes were calculated from the 3D dataset by identifying the mitral annulus and apex at end-diastole and end-systole.[7] An automated (manually-adjustable) endocardial edge detection system calculated the LV end-diastolic (LVEDV) and end-systolic (LVESV) volumes. This dataset was used to provide 3D ejection fraction (EF) and also to detect LV epicardial boundaries to derive LV mass. The LV end-diastolic major axis from the 3D dataset was used to calculate a 3D sphericity index.[10] 2D ejection fraction (EF) was also calculated (using the Simpson’s biplane method), but the 3D EF was used preferentially in data analysis, as it makes no assumptions about LV shape [7]; a factor that is particularly important given the observations of LV remodelling in pregnancy [12–14]. For the same reason, 3D (but not 2D) EF was used in the multivariate linear regression analysis to determine predictors of LV remodelling.

LV 2D strain analysis was performed using Qlab advanced quantification software version 9 (Philips, MA). Speckle tracking was performed using the software’s automated edge detection system, and adjusted manually as necessary.[11] Analysis was only performed on images of sufficient quality for speckle tracking to be performed accurately throughout the cardiac cycle. Longitudinal strain was obtained in the LV 4 chamber, 2 chamber and 3 chamber views and global longitudinal strain (GLS) was calculated by combining these measurements.

### Biomarkers

Blood samples were aliquoted, frozen and batch analysed in a blinded fashion. We quantified adrenomedullin, high-sensitivity C-reactive protein (hsCRP), NT-pro-brain natriuretic peptide (NT-pro-BNP), oxidized low density lipoprotein (ox LDL), placental growth factor (PlGF) and soluble fms-like tyrosine kinase 1 (sFlt1).

### Statistics

Subject characteristics are summarised as mean±SD and other results as mean±SEM. Data were tested for normality and compared using either one-way ANOVA or the Mann-Whitney U test, as appropriate. Ethnicity distribution was assessed by the chi-squared test for categorical variables. Pearson correlation coefficients were used to identify linear relationships between continuous variables. Independent predictors of LV remodelling were identified by multivariate linear regression. All tests were 2-tailed and differences were considered significant at p<0.05. Bonferroni correction was applied for multiple comparisons.

Measurement variability for 3D data and 2D strain was assessed in fifteen randomly selected studies using intra-class correlation coefficients. Intraclass correlation coefficients demonstrated inter-observer variability of r = 0.84 for 3D EF, r = 0.73 for GLS. Intra-observer variability was r = 0.75 for 3D EF, r = 0.79 for GLS.

## Results

### Baseline characteristics

The control and healthy PP groups were matched for age, ethnicity and BMI and there were no differences between the subgroup of 40 PP women who underwent 3D echocardiography and the combined group of 58 PP women (Table 1). Healthy PP women had significantly lower systolic, diastolic and mean arterial blood pressures than controls.

**Table 1:** Baseline characteristics of control and healthy postpartum groups. Healthy PP women had significantly lower systolic, diastolic and mean arterial blood pressures than controls. Data are presented as mean ± SD. BMI, body mass index; BSA, body surface area; Healthy PP, healthy postpartum; MAP, mean arterial pressure; PPCM, peripartum cardiomyopathy; *p<0.001, compared to controls.

Characteristic	Control	Healthy PP	Healthy PP
		*Whole group*	*3D echo*
n	18	58	40
**Ethnicity n (%)**			
*Afro-Caribbean*	9 (50)	26 (45)	19 (48)
*Caucasian*	9 (50)	32 (55)	21 (52)
**Age (years)**	32.1±7.2	31.1±5.6	31.8±4.8
**BMI (kg/m**^**2**^**)**	25.1±5.2	27.2±6.3	27.0±5.5
**BSA (m**^**2**^**)**	1.74±0.2	1.83±0.2	1.83±0.2
**Systolic BP (mmHg)**	121.4±15.6	110.4±10.3	109.3±10.1*
**Diastolic BP (mmHg)**	78.6±7.2	69.3±8.6	68.8±8.9*
**MAP (mmHg)**	91.8±9.9	83.1±8.4	82.5±8.5*

The PPCM group had a mean age of 35.0 ± 2.4 years and a similar distribution of ethnicity as the control and healthy PP groups. Values for systolic blood pressure (113.9±20mmHg, p = 0.27) and diastolic blood pressure (73.0±15mmHg, p = 0.18) in PPCM patients were similar to healthy controls.

### Left ventricular structure

**Fig 1:**
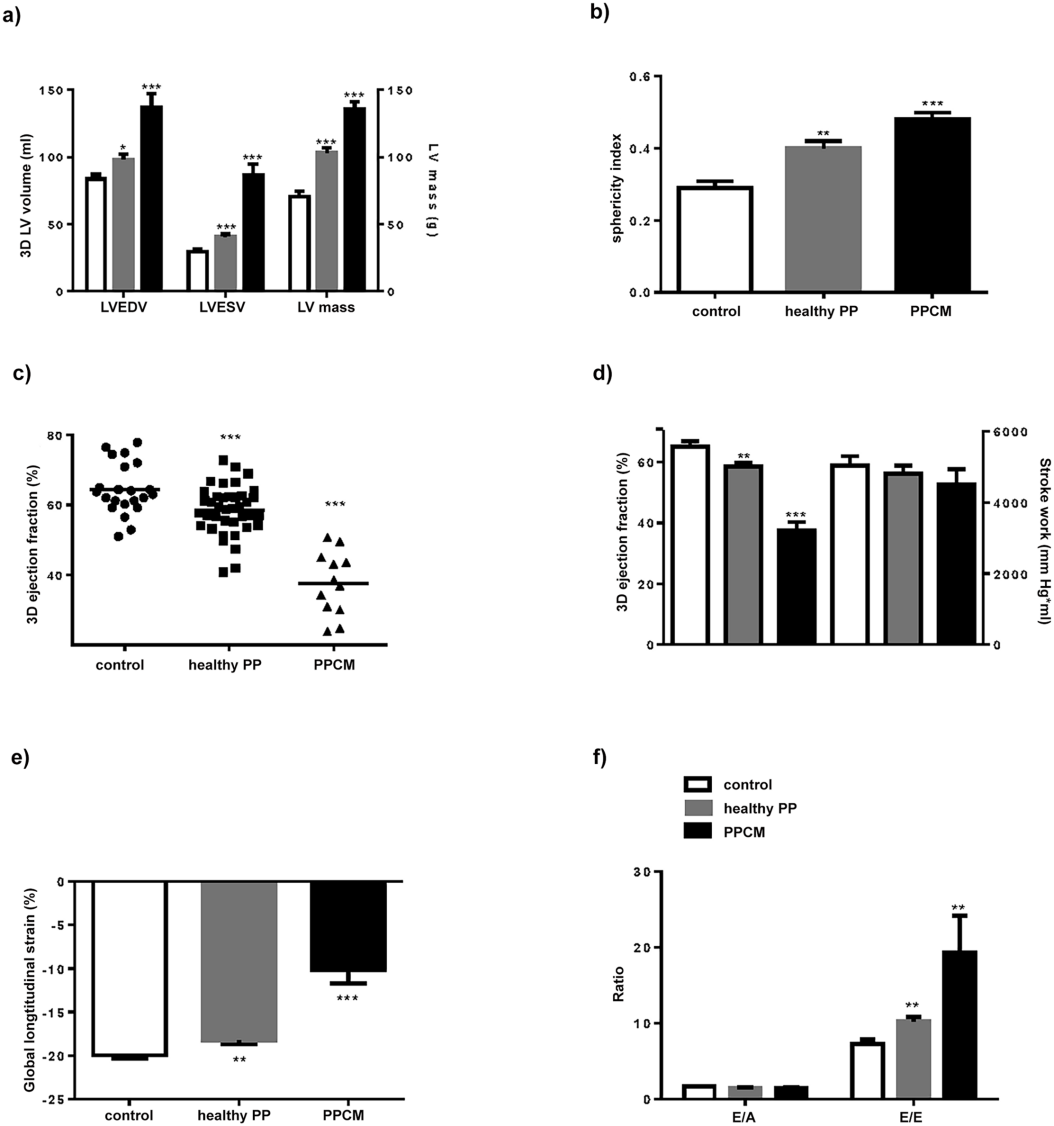
Echocardiographic indices in control, healthy postpartum and postpartum cardiomyopathy groups. a) LV volumes and mass, b) sphericity index, c) 3D ejection fraction, d) 3D ejection fraction vs. stroke work, e) global longitudinal strain, and f) diastolic function. LVEDV, LV end-diastolic volume; LVESV, LV end-systolic volume. *p<0.05, **p<0.01, ***p<0.001.

Healthy PP women had significantly larger LVEDV, LVESV, mass and sphericity index than controls by 3D echocardiography (Fig 1a and 1b). The LV mass/LVEDV ratio was also significantly higher in the PP group (1.04±0.45 vs. 0.85±0.05 in controls, p = 0.01), suggestive of concentric hypertrophic remodelling. These structural changes were more exaggerated in PPCM patients (Fig 1a and 1b).

### LV systolic and diastolic function

Healthy PP women had a significantly lower 3D ejection fraction (EF) than controls (58.5±1.1 vs. 65.0±1.9%, p<0.01) but LV stroke work was unchanged **(**Fig 1c and 1d**)**. GLS was also significantly lower in the postpartum group (Fig 1e). In parallel with structural changes, reduction in LV systolic function was more pronounced in PPCM patients than healthy PP women (Fig 1c and 1d). Healthy PP women and PPCM patients had significantly raised E/E’ ratio compared to controls (Fig 1f).

### Biomarkers

**Fig 2:**
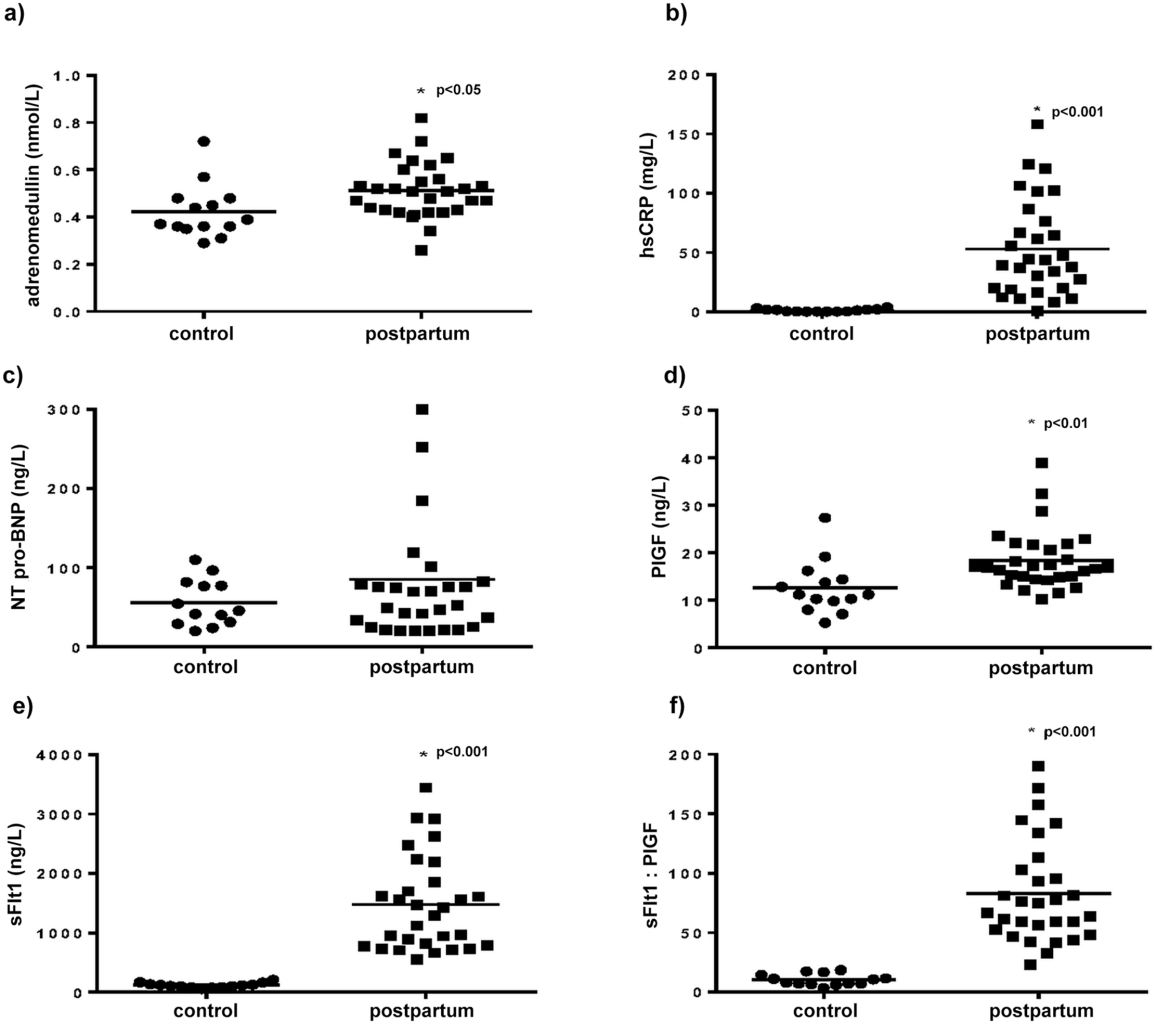
Serum levels of cardiac biomarkers and markers of oxidative stress in control vs. healthy postpartum women. a) adrenomedullin, b) NT-pro-BNP, c) hsCRP, d) ox-LDL, e) sFLT-1, f) PlGF, g) sFlt1:PlGF. hsCRP, high sensitivity C-reactive protein; NT-pro-BNP, N-terminal pro-brain natriuretic peptide; ox-LDL, oxidised LDL; PlGF, placental growth factor; sFlt1, soluble fms-like tyrosine kinase.

Healthy PP women had higher levels of adrenomedullin, hsCRP, sFlt1 and PlGF than matched control subjects (Fig 2). The ratio of sFlt1/PlGF was also higher in healthy PP women. There were no differences between the control and postpartum groups in levels of NT-pro-BNP or oxidised LDL (19.1±6.0 vs. 13.1±3.1μg/L, respectively; p = 0.34).

### Repeat echocardiography in women with reduced ejection fraction

The lowest value for 3D EF in the control group was 51%. 6 healthy PP women had a 3D EF ≤ 50% (range 40.8–49.7%). None of these had any symptoms or signs of cardiovascular dysfunction. These 6 women attended for repeat echocardiography 4 to 8 months after their initial studies, by when EF had increased from 47.4±0.34 to 60.8±0.18%. Although the number of subjects was small, several biomarkers in this subgroup were higher than those in the overall healthy PP group (adrenomedullin 0.54±0.1 vs. 0.51±0.02 pg/ml; hsCRP 72.1±2.9 vs. 52.9±7.4 mg/L; NT-pro-BNP 135±4.0 vs. 85.3±1.8 pg/ml).

### Ethnic differences

Healthy PP Afro-Caribbean and Caucasian women had similar ages, BMI and blood pressure (Table 2). LV volumes, mass and sphericity index were similar between the groups. There was a trend towards lower stroke work index in Afro-Caribbean vs. Caucasian women, but EF was not significantly different. The E/A ratio was significantly lower in Afro-Caribbean women. Afro-Caribbean women had significantly lower values than Caucasian women for strain in two of the three apical segments but GLS was similar between the groups (Table 2). All measured biomarkers were similar between the groups (data not shown).

**Table 2:** Comparison of ethnic variations in LV remodelling and function. Compared to Caucasian women, Afro-Caribbean women had a lower E/A ratio and reduced longitudinal strain in two apical LV walls. AP3 APA, apical three-chamber apical anterior; AP2 BA, apical two-chamber basal anterior; AP4 MAL, apical four-chamber mid-anterolateral; BMI, body mass index; GLS, global longitudinal strain; 3D LVEDV, left ventricular end-diastolic volume, 3D LVESV, left ventricular end-systolic volume; 3D EF, ejection fraction.

Parameter	Afro-Caribbean	Caucasian	p value
**Age (years)**	30.2±5.5	31.8±5.6	0.29
**BMI (kg/m**^**2**^**)**	28.2±6.1	26.5±6.5	0.32
**Systolic BP (mmHg)**	110.7±10.2	109.6±10.7	0.70
**Diastolic BP (mmHg)**	68.7±7.6	69.6±9.4	0.69
**LVEDV (ml)**	93.5±4.5	103.2±4.6	0.22
**LVESV (ml)**	40.3±2.1	40.6±2.5	0.94
**LV mass (g)**	100.7±4.1	105.7±4.3	0.52
**Sphericity index**	0.72±0.03	0.38±0.02	0.49
**3D EF (%)**	56.7±1.50	60.2±1.67	0.12
**Stroke work index (g/cm**^**2**^**)**	46.4±1.78	49.9±1.39	0.13
**E/A ratio**	1.56±0.07	1.37±0.06	0.046
**E’ lateral**	8.31±0.69	11.2±1.1	0.063
**Strain**			
*GLS*	-18.4±0.8	-18.5±1.0	0.91
*AP4 MAL*	-17.6±1.26	-22.6±1.20	0.009
*AP2 BA*	-15.1±2.49	-22.8±2.15	0.039
*AP3 APA*	-14.3±1.51	-18.8±1.80	0.07

### Potential determinants of LV remodelling and function

We investigated the correlation between three key echocardiographic parameters of LV remodelling/function (i.e. LV mass index, LVEDVI and EF) and clinical characteristics and cardiac biomarkers in the combined group of healthy PP women and controls **(**Table 3**)**. The postpartum state had a significant positive association with LV mass index and LVEDVI and a significant negative association with 3D EF. LV mass index correlated negatively with MAP and positively with adrenomedullin, hsCRP and sFlt-1.

**Table 3:** Pearson’s correlation coefficients for clinical characteristics and cardiac biomarkers with a potential effect on echocardiographic parameters of LV remodelling in healthy postpartum patients and controls. The postpartum state was negatively associated with 3D ejection fraction, and positively associated with LV mass index and LVEDVI. LV mass index correlated negatively with MAP and positively with adrenomedullin, hsCRP and sFlt-1. Adrenomed, adrenomedullin; hsCRP, high sensitivity C-reactive protein; NT-pro-BNP, N-terminal pro-brain natriuretic peptide; PlGF, placental growth factor; PP state, postpartum state, Flt1, soluble fms-like tyrosine kinase.

	LV mass index			3D EF			LVEDVI	
	r	P	B±SE	r	P	B±SE	r	P
**Age**	-0.11	0.44		-0.04	0.74		0.09	0.44
**Ethnicitiy**	-0.12	0.41		-0.21	0.12		-0.01	0.91
**PP state**	0.51	<0.001	12.7±3.4^‡^	-0.39	<0.01	5.0±1.6†	-0.15	0.20
**MAP**	-0.31	0.03		0.05	0.75		-0.04	0.73
**Adrenomed**	0.45	<0.01	56.1±20.8^†^	-0.31	0.07		-0.06	0.73
**hsCRP**	0.39	0.02		-0.27	0.12		-0.12	0.45
**BNP**	0.13	0.45		-0.19	0.29		-0.07	0.69
**sFlt1**	0.36	0.03	0.007±0.003*	-0.30	0.08		-0.14	0.37
**PlGF**	0.29	0.09		-0.12	0.48		0.13	0.41
**sFlt1:PlGF**	0.32	0.06	0.13±0.07	-0.29	0.09		-0.29	0.07

*p<0.05

^†^p<0.01

^‡^p<0.001

In multivariate linear regression analysis **(**Table 4**)**, adrenomedullin was an independent predictor of LV mass index (p<0.03), and the postpartum state was an independent predictor of 3D EF (p<0.02). sFlt1 and sFLT1:PlGF were independent predictors of LV end-diastolic volume index (p<0.01 for both).

**Table 4:** Independent predictors of LV mass, 3D ejection fraction and LV end-diastolic volume index. The postpartum state was an independent predictor of 3D ejection fraction. sFlt1 and sFLT1:PlGF were independent predictors of LV end-diastolic volume index, and adrenomedullin was an independent predictor of LV mass index. PlGF, placental growth factor; PP state, postpartum state, Flt1, soluble fms-like tyrosine kinase.

Parameter	B±SE	P	95% CI
**LV mass index**			
***Adrenomedullin***	51.7±22.8	0.03	5.17 to 98.2
**3D EF**			
***PP state***	-6.55±2.60	<0.02	-11.9 to -1.2
**LVEDV index**			
***sFlt1***	0.028±0.009	<0.01	0.007 to 0.046
***sFLT1*:*PlGF***	-0.059±0.18	<0.01	-0.95 to -0.22

## Discussion

It is well established that pregnancy is accompanied by significant changes in systemic haemodynamics and structural cardiac remodelling.[12] Cardiac output, stroke volume and heart rate increase significantly in the 3^rd^ trimester,[4, 12] while the LV undergoes dilatation and eccentric hypertrophy.[13–15] The changes in LV mass, wall stress and longitudinal strain normalise by 2 weeks to 6 months postpartum.[15, 16] However, little is known about cardiac function during the early postpartum phase- a period of large and relatively rapid changes in maternal haemodynamic state—with previous studies focusing on women no earlier than 2 weeks post-partum.[16]

In this study, we characterise using advanced 2D and 3D echocardiography the normal physiological range of cardiac size and function in Caucasian and Afro-Caribbean women in the early postpartum period. By studying only women with uncomplicated vaginal delivery in the healthy postpartum group, we avoided any confounding effect of surgery. The most striking finding of our study is that LV systolic function is mildly but significantly reduced in healthy postpartum women as compared to matched non-pregnant controls. A quarter of healthy postpartum women have an EF<55%, falling into a range that would normally be described as “mildly impaired” outside the peri-partum period.[17] In addition, healthy post-partum women also had significantly reduced GLS, a relatively load-independent parameter of systolic function. The finding of raised E/E’ ratios at the mitral annulus may reflect increased fluid load rather than true diastolic dysfunction. However, none of the women with reduced EF had any symptoms or signs of cardiovascular dysfunction, and those with the most markedly reduced EF who attended for repeat echocardiography 6 months later showed complete normalization of LV function without the need for any specific therapy. Furthermore, levels of NT-pro-BNP were not significantly different between healthy postpartum women and controls and there was no evidence to suggest that these subjects had sub-clinical PPCM. Taken together, these data suggest that the reduced LV systolic and diastolic function observed in these healthy postpartum women represent part of the normal adaptive physiological response to pregnancy rather than a pathological phenomenon.

Our retrospective analysis of patients with PPCM, who had a 3D echocardiogram at the time of diagnosis, allowed comparison with control and healthy postpartum women. As expected, patients with PPCM had significantly impaired LV systolic and diastolic function and increased LV volumes. When compared to healthy postpartum women as a group, however, these changes were substantially greater in magnitude. Nevertheless, it is of interest that there was a degree of overlap in EF between healthy postpartum women with the lowest EF values and PPCM patients. This finding is of particular clinical relevance in the context of making a diagnosis of PPCM in the early postpartum period. In apparently healthy women without any cardiovascular symptoms, we concluded that slight reduction in LV systolic function immediately post-partum was part of the normal physiological adaptation to pregnancy, but we recognise that this is a somewhat arbitrary classification. It remains possible that some of the “healthy” women who demonstrated slight reduction in LV function may have had minor pathological impairment that recovered without specific treatment. This overlap between overt mild PPCM and apparently normal post-partum LV function is important to bear in mind when assessing women in the post-partum period. Information on pre-delivery cardiac function would clearly be valuable in such cases.

We employed 3D echocardiography to quantify LV volumes and EF, a method that correlates well with MRI;[7] to the best of our knowledge, all prior studies on post-partum women used 2D echocardiography. Previous work on cardiac adaptation during pregnancy reported either no changes in EF[15] or a small decrease in the last trimester compared to 6 months postpartum,[18] but these studies used the Simpson’s biplane method to estimate EF. The use of speckle tracking to quantify myocardial deformation provided the advantages of an angle-independent and relatively load-independent technique. Our finding of reduced GLS in the healthy postpartum group mirrors previously described changes in the last trimester.[15, 18, 19] This reduction in LV strain is thought to be a consequence of increased LV dimensions, requiring less shortening to produce the same stroke volume. GLS is reported to normalise at 3–6 months postpartum.[15] The LV sphericity index was significantly increased in postpartum women compared to controls, a change that in a disease setting could suggest pathological LV remodelling. Previous studies described an eccentric pattern of LV hypertrophy during pregnancy,[15] but it should be noted that they employed the relative wall thickness/chamber size method whereas the 3D method makes no assumptions about the shape of the LV.[7] LV end-diastolic filling pressures were elevated in healthy postpartum women, as reflected by an increased E/E’ ratio, with a more pronounced change in patients with PPCM.

Maternal ethnicity may influence LV systolic and diastolic function[8] and also influences the incidence of PPCM.[1] We therefore studied variations amongst the two main ethnic groups in our population. Values for strain and diastolic function parameters differed somewhat between Afro-Caribbean and Caucasian women. However, both groups showed similar reductions in LV systolic and diastolic function as compared to matched non-pregnant controls, implying that the basic physiological changes occurring in the early postpartum stage are not influenced by ethnicity. Of note, ethnicity did not influence the presence of persistent LV trabeculations in healthy postpartum women in a recent study.[20]

Although there was no difference in NT-pro-BNP between healthy postpartum women and controls, the postpartum group had significantly higher levels of sFlt1, PlGF, adrenomedullin and hsCRP. sFlt1 is an anti-angiogenic factor that binds competitively to the pro-angiogenic placental growth factor (PlGF), and the ratio of sFlt1/PlGF reflects the balance of anti- to pro-angiogenic factors. This ratio has diagnostic and prognostic value in pre-eclampsia.[21] Furthermore, sFlt1 levels were reportedly raised in women with PPCM compared to controls at 6 weeks postpartum, and this was suggested to be pathogenically important in a mouse model of the condition.[22] Adrenomedullin is a potent vasodilatory hormone, levels of which are elevated in congestive heart failure.[23] Its levels also rise progressively through pregnancy and decrease rapidly to non-pregnant levels within 4 days postpartum.[24] In our study, adrenomedullin was an independent predictor of LV mass index while sFlt1 and sFLT1:PlGF were independent predictors of LV end-diastolic volume index. Since these are biomarkers of preeclampsia, and pre-eclampsia may increase the risk of PPCM,[25] it is interesting to speculate that there may be some overlap in the underlying mechanisms that are responsible for cardiac remodelling in the “normal” post-partum period and those that drive PPCM. However, the mechanistic basis for these associations, if any, requires more extensive study. Our finding of unchanged NT-pro-BNP levels between the postpartum and control group agrees with the work of Nanno et al,[26] but differs from another report that BNP rises in the first 72 hours after delivery.[27] Differences in the assays used may account for this discrepancy. It should be noted, however, that the spread of NT pro-BNP values was greater in the postpartum group than controls. We measured oxidised LDL levels as a potential readout of increased oxidative stress associated with pregnancy,[28] but no significant differences were found between healthy postpartum women and controls. The increase in hs-CRP found in postpartum women is consistent with prior reports of an increase during pregnancy.[29]

In summary, this study identifies the occurrence of a significant physiological reduction in LV systolic and diastolic function in healthy postpartum women as compared to age- and ethnicity-matched controls. Healthy postpartum women also have increased levels of anti-angiogenic and inflammatory markers in the first 48 hours after delivery. Although patients with PPCM had substantially worse LV systolic and diastolic dysfunction, the overlap between groups in EF indicates that particular care is needed to confidently diagnose mild PPCM is the early post-partum period. However, healthy postpartum women are expected to show a spontaneous increase in EF in the weeks after delivery. It would be important to further explore factors that can differentiate physiological adaptation from PPCM-induced changes, as has been demonstrated recently for hypertensive heart failure of pregnancy.[30] It would also be of value to re-assess strain in patients with PPCM during follow-up visits, in order to evaluate its potential as a prognostic marker.

### Study limitations

Our study had a relatively small sample size although the groups were well matched. Ideally, the study would have included an additional control group comprised of normal term pregnancy. However, it was not possible for logistic reasons to undertake a serial study of changes in cardiac function and the analyses therefore relied on comparisons between postpartum women and matched controls. We measured longitudinal strain, as this has been shown to decrease during pregnancy and normalise postpartum, with radial and circumferential strain undergoing no significant change.[15] However, the measurement of radial and circumferential strain, as well as strain rate, might provide additional information on the nature of the LV remodelling. The group of PPCM patients was a retrospective sample from our institution and we did not have data available for changes in cardiac biomarkers in this cohort.
